# Effect of economic development, income inequality, transportation, and environmental expenditures on transport emissions: evidence from OECD countries

**DOI:** 10.1007/s11356-022-19580-6

**Published:** 2022-03-26

**Authors:** Zahid Hussain, Muhammad Kaleem Khan, Wasim Abbass Shaheen

**Affiliations:** 1 School of Finance, Qilu University of Technology (Shandong Academy of Sciences), Jinan, China; 2 Asia-Australia Business College, Liaoning University, Shenyang, China; 3 School of Management Sciences, Quaid-i-Azam University, Islamabad, Pakistan

**Keywords:** Transportation, EKC, Environmental expenditures, Economic development, OECD

## Abstract

Environmental quality has been pondered as an essential aspect of sustainable development across the global economies. Several factors such as economic development, income inequality, transportation, and environmental expenditures drastically influence environmental quality. More specifically, the transport sector is a major contributor to carbon emissions which deteriorate the environmental quality. Therefore, this study investigates whether economic development, transportation, environmental expenditures, and income inequality affect transport-carbon emissions for the OECD countries. Furthermore, panel time-series data period from 2000 to 2020 and cross-sectional autoregressive distributed lag method are used for transport-oriented environmental examination. Results demonstrate that transportation upsurges transport-carbon emission level by 46.45% on average. Moreover, the joint effect of economic development and environmental taxes significantly reduces transport-carbon emissions by 14.70%. Findings further suggest that an inverted *U*-shaped relationship exists between economic development and transport emission. Besides, income inequality, environmental expenditures, and green transportation are negatively associated with the coefficient of transport-carbon emissions. More interestingly, income inequality is negatively correlated with transport-carbon emissions across the sample countries. Furthermore, the joint effect of income inequality and economic development increases the emission level released by the transport sector. Thus, this research recommends some policies: countries should control traffic movements and increase environmental expenditures, and produce green transport vehicles to tackle environmental issues.

## Introduction

Environmental concentrations have progressively augmented since the occurrence of industrial revolution (Khan et al. 2021a). Global temperature gradually increased due to rapid level of economic development and complex transportation systems of the countries. Therefore, environmental quality became a challenging issue at the globe, which deteriorate due to carbon dioxide (CO_2_) emissions (Ahmad et al., 2021). Transportation sector is a major source of CO_2_ emissions approximately 23% of the total CO_2_ emissions (Sohail et al. 2021; Seum et al. 2020; Churchill et al. 2021). Global CO_2_ emissions from the transportation sector will be increased approximately 60% by 2050 in the absence of effective mitigation strategies (ITF 2019). According to the International Energy Agency (IEA), the transportation sector accounts for around one-third of worldwide CO_2_ emissions. Several economic activities, such as broad-scale transportation infrastructure development, vehicle traffic movement, population expansion, and economic growth, have increased demand for transportation vehicles and caused serious dangers to long-term development. For instance, construction of roads, railways, and airports require an increasing number of resources, such as land area, technology, energy resources, development of urbanization, industrialization, and economic externalities, which directly and indirectly reduce greener land and release greenhouse emissions. Consequently, environmental quality is deteriorated over time (Khan et al. 2021b).

Traffic also causes the problem in the transportation sector and the environment. A high volume of passenger and freight traffic results in a trillion journeys every year and increases the demand for locomotives (vehicles). CO_2_ emissions are significantly increased by these movements and locomotives (Hadavi et al. 2020). Verlinghieri (2020) demonstrated that traffic movements indirectly impact CO_2_ emissions due to the use of vehicle engines on roads and railways. Similarly, Heinold (2020) presented that road and railway transport emits a significant quantity of carbon emissions due to energy use.

Several potential studies on transportation modes in terms of CO_2_ emissions have been extensively investigated. This study aims to answer the following research questions: Does transportation effect the CO_2_ emissions (road, rail, and air)? According to the Organization for Economic Cooperation and Development, OECD countries present high-income economies that account for approximately 42.8% of global GDP at purchasing power parity (PPP). These countries are expected to consume an additional 1.4% of their total transportation energy consumption each year (from 104 quadrillion Btu to 155 quadrillion Btu) in the period of 2012–2040. How economic development and income inequality affect transport-carbon dioxide emissions? Do environmental expenditures and green transportation impact CO_2_ emissions?

This analysis focuses on OECD countries due to some reasons. First, the OECD countries are high-income economies that remarkably contribute to the global economy at around 42.8% of GDP at purchasing power parity. Second, the OECD countries are responsible for CO_2_ emissions and their transportation energy consumption is expected to increase annually by 1.4% between 2012 and 2040 (from 104 quadrillion Btu to 155 quadrillion Btu). Furthermore, the OECD and non-OECD countries consume approximately 55% and 45% of global transport energy, respectively (IEA 2019). Third, these countries are knowledge-based economies. Billions of passengers and freight journeys every year result in traffic movements, and they are usually the target of traffic movements for international economic processes. Fourth, they are committed to reducing emissions and enhancing environmental quality because they allocate a high portion of their GDP to the reduction of CO_2_ emissions. Finally, these countries typically transfer their transportation resources away from carbon-based modes and toward green modes (e.g., electric vehicles). Although CO_2_ emissions may be caused by several factors, the impact of transportation energy consumption on the relationship between CO_2_ emissions and traffic movements, locomotives, and environmental government budget remains unverified. Traffic movements and green transportation must be considered when developing sustainable transportation policies because they exert a significant impact on CO_2_ emissions in OECD countries.

This study contributes the following. First, linear and nonlinear influences of GDPPC on transportation CO_2_ emissions (TCO_2_) are examined. Second, the joint effect of economic development and environmental taxed on TCO_2_ is investigated. Besides, the joint effect of income inequality and economic development is also examined. Third, a new insight into the link between TCO_2_ and transportation (road, rail, and air) is undertaken. Fourth, the impact of environmental research and development expenditures (ERDE) on CO_2_ emissions and the joint influence of ERDE and environmental taxes on CO_2_ are explored. Fifth, whether green transportation employs a major impact on TCO_2_ is determined. Finally, the effect of traffic movements, transport energy consumption, ERDE, and green transportation on TCO_2_ is assessed using the cross-sectional autoregressive distributed lag (CS-ARDL) model.

The remainder of this paper is organized as follows. A summary of previous studies is presented in the “Literature review” section. The theoretical foundation, data source, and technique are presented in the “Methodology” section. Empirical findings and interpretations are discussed in the “Empirical results and discussions” section. The results of this study and policy implications are provided in the “Conclusion” section.

## Literature review

Several empirical studies emphasized on the relationship between environment, economic development, and transport across the emerging, developed, and developing countries. The main focal point is whether economic development, transport, and expenditures have significant effect on environment quality. Thus, current study summarizes the previous findings on the relationship between economic development, traffic, environmental expenditure, and transport-carbon emissions. Therefore, environment and transportation may be investigated in multiple aspects. Expansion in transportation sector has been commonly augmented the cost of environmental quality. Impacts of the transportation industry on the environment differ depending on transportation techniques and legislation.

### Nexus between environment and transportation

This strand of the literature emphasizes on the relationship between the environment and transportation that has been recently received much concerned. For instance, Churchill et al. (2021) investigated the impact of transportation infrastructure on CO_2_ emissions for a panel of OECD countries using parametric and nonparametric techniques. CO_2_ emissions increase by 0.4% for every 1% increase in transportation infrastructure. The nonparametric estimate also showed a time-varying relationship between transportation infrastructure and CO_2_ emissions, which has been positive since World War II. More critically, transportation infrastructure (such as road, rail, and airport building) is not the main source of CO_2_ emissions. Emissions are also heavily influenced by traffic activity. Consequently, this study examines the impact of traffic operations (such as movement and locomotives) on emissions from the transportation industry. Subsequently, Churchill et al. (2021) concentrated on the link between road transportation and CO_2_ emissions in 22 European nations and demonstrated that the transportation sector emits around 27% of total CO_2_ emissions. Furthermore, (fossil) fuel usage is nearly exclusively responsible for road TCO_2_. As a result, energy usage, particularly in the transportation sector, is a very important predictor of CO_2_ emissions. According to Pani et al. (2021), freight transportation increases greenhouse emissions in the largest countries of the world because truck vehicles used to transport goods increase energy demand. Hence, generated CO_2_ emissions degrade the quality of the environment. Similarly, Cardenete and López-Cabaco (2021) showed that cargo transportation is a highly effective component, with nearly 30% of all forms of transportation contributing to CO_2_ emissions in Spain. Arvin et al. (2021) investigated the relationship between TCO_2_ and energy use in Germany. The fuel used in transportation vehicles (such as gasoline and diesel) increases CO_2_ emissions through traffic locomotives. Umar et al. (2021) focused on the impact of biomass and fossil fuel energy consumption on CO_2_ emissions from the transportation sector in the USA and demonstrated that fossil fuel energy exerts a significant and positive impact on CO_2_ emissions from the transportation sector. However, a negative correlation exists between the use of biomass energy and CO_2_ emissions. The relationship of CO_2_ emissions and energy use presents an inverted *U*-shape.

Hussain et al. (2020a, b) argued that there is a negative relationship between climate change potential (CO_2_ emissions) and transportation infrastructure although development infrastructure is a major source of CO_2_. Notably, catastrophic climate change can potentially limit transportation operations through fundamental infrastructures. Subsequently, Ahmad et al. (2021) also investigated the transport emission effects that energy use releases the emissions through the transportation sector at the atmosphere, which deteriorate the environmental quality. Furthermore, Sohail et al. (2021) emphasized the link between green transportation and the environment for reducing CO_2_ emissions, which reveal that using the green transportation is the optimal effective way of minimizing the CO_2_ emissions. Electric vehicles reduce the demand for fossil fuels while boosting the demand for electricity. However, resources are diverted from fossil fuels to electric cars because electricity shortages occur in the market. Consequently, CO_2_ emissions have tended to declination from the transportation sector. Another study, e.g., Oryani et al. (2021), demonstrates that green transportation can be a better strategy for cutting the CO_2_ emissions by promoting renewable electric cars to minimize CO_2_ emissions per capita, shifting the internal combustion engine cars to alternative fuel vehicles, which can result in significant CO_2_ reductions.

### Nexus between environment and expenditures

This strand of the literature focuses on some studies, which have shown that environmental taxes can be used to reduce CO_2_ emissions. Bergantino et al. (2021) investigated the impact of taxes on CO_2_ emissions to enhance environmental quality and suggested that automobile levies reduce CO_2_ emissions because demand and supply in the market diminish at the same time. As a result, the increase of automobile sharing decreases CO_2_ emissions. According to Khastar et al. (2020), a suitable environmental tax level exerts a moderating impact on CO_2_ emissions in European Union countries. Reaños (2020) also exhibits that carbon taxes have a considerable impact on CO_2_ emissions. Carbon taxes (at least 30 Euros per ton of CO_2_ emissions) must be levied on vehicle owners to reduce CO_2_ emissions. Additional carbon taxes based on price elasticities may encourage vehicle owners to consume additional energy. The impact of environmental R&D on CO_2_ emissions from the transportation industry is also considered in the literature. A wide-ranging investigation has shown that R&D helps reduce CO_2_ emissions and enhance environmental quality. In this regard, Petrović and Lobanov (2020) indicated that R&D in OECD nations exerts a negative impact on CO_2_ emissions on average. An R&D spending increase of 1% results in a 0.15% reduction in CO_2_ emissions on average. Increasing R&D spending reduces CO_2_ emissions; however, their findings do not apply to 40% of nations due to resource constraints. Wang and Zhang (2020) presented that a 1% increase in R&D spending in BRICS nations decreases CO_2_ emissions by 0.8122%.

### Nexus between environment and economic development

This strand of the literature sheds light on the relationship between economic development and environment. Several studies, e.g., Zhang and Zhang (2021), argued that level of economic development has remarkable impact on carbon emissions in inclination trend. In the contrast, income inequality is negatively associated with carbon emissions through marginal propensity to consume concern over consumers. Likewise, Wang et al. (2020a, b, c) and Wang et al. (2020a, b, c) examined the relationship between transport-carbon emissions and economic development. They quantified the effect of economic decoupling on environment. Their findings reveal that transport-carbon intensity overall declined due to polarization trend across the panel. Transport-carbon emissions are influenced by expansive decoupling, recessive decoupling, and weak decoupling over time. Afterward, environmental degradation is substantially reduced due to economic development in the Belt and Road countries. Moreover, Wang et al. (2021a, b) and Wang et al. (2020a, b, c) discussed economic development causes carbon emissions through transport infrastructure development. Transport infrastructure construction also becomes source of carbon emissions during the construction activities such as energy use and transport via fossil fuel, which indicate the economic development process. In addition, Song et al. (2021) argued that economic growth increases the carbon emissions. More specifically, a continued rise in economic growth requires more and more natural resources to produce consumer and producer goods, which cause the environmental deterioration through releasing the carbon emission at the atmosphere. Another study, e.g., Hussain (2022), measures the economic-oriented environmental performance by using economic growth. Environment is strongly influenced by economic growth although it produces positive externalities. However, it reduces the environmental quality by polluting the atmosphere.

This research fills the gap by investigating the joint effect of economic development and income inequality, and environmental taxes including the expenditures related to environment. Previous studies did not consider the simultaneous use of economic development and income inequality including environmental taxes and also nonlinear effect. Investigation on the relationship between traffic movements and vehicles is not undertaken at broad perspective. Furthermore, the combined impact of transportation energy consumption and traffic locomotives remains unproven. Previous studies also snubbed the nonlinear effect of transportation energy usage. Moreover, the relationship between TCO_2_ and traffic, simultaneous use of economic development, income inequality and environmental taxes, ERDE, and green transportation usually ignores possible heterogeneity and cross-sectional dependency. Consequently, the existing literature encloses the significant gap and most relevant approaches, which should be used to investigation on environment-oriented transport sector.

## Methodology

### Theoretical framework

**Fig. 1:**
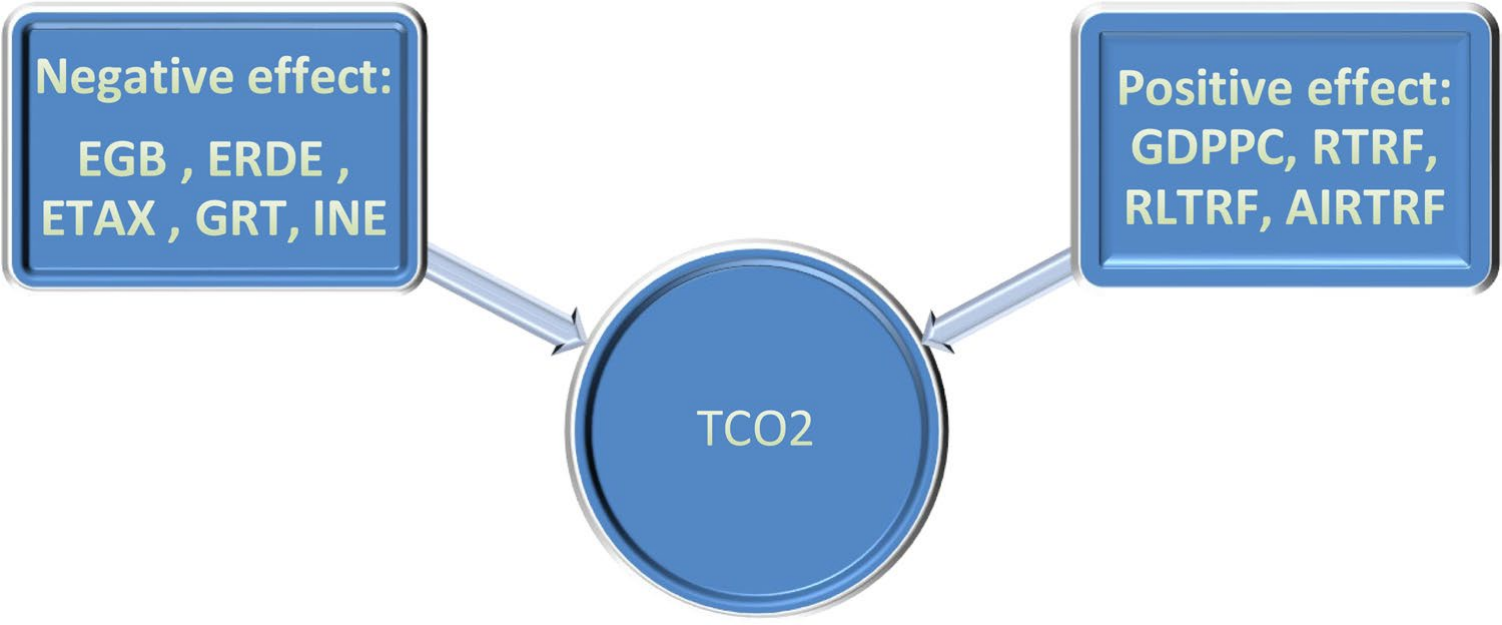
theoretical framework

Figure 1 illustrates that traffic, environmental expenditures, green transport, income inequality, and economic development are significantly associated with transport CO_2_ emissions. Therefore, environmental Kuznets curve (EKC) theory reveals that economic development requires additional natural resources at the initial level. Economic development upsurges aggregate demand for inputs to produce a certain level of outputs from all sectors of the economy. More specifically, the transport sector significantly contributes to economic development through the construction of transport infrastructure, traffic, and energy consumption under this scenario. The demand for transport energy increases with the increase of economic development and causes greenhouse emissions in the atmosphere. Consequently, emissions, such as carbon dioxide emissions released by transport sector and produced wasteful material, pollute the environment. However, the manufacturing process uses the greenhouse emissions and pollution substantial for the specific sector (Ahmad et al. 2020). Thus, the structural transformation is predicted to reduce damaging effects of economic development on climate change.

Moreover, economies adopt advanced technologies that increase the income level and improve productivity and environmental quality (Zhang and Zhang 2021; Liobikiene and Butkus 2019; Ahmad et al. 2020). Besides, income inequality also affects the carbon emission. For instance, a higher income class has lower rate of marginal propensity to consume regarding the commodities which stimulate the emissions through consumption channels. In the contrast, lower income class has positive effect on carbon emissions. The reason may be behind that a lower income class can have higher rate of marginal propensity to consumer. More specially, lower income class has tendency to consume the commodities for positive utility. Under such condition, aggregate demand for the goods is increased, which require the natural resources for usage, and pollute the environment.

1 $${\text{TCO}2}_{\text{it}}={\beta}_0+{{\beta}_1\left({\text{GDPPC}}_{\text{it}}\right)+\beta}_2\left({\text{RTRF}}_{\text{it}}\right)+{\beta}_3\left({\text{RLTRF}}_{\text{it}}\right)+{\beta}_4\left({\text{AIRTRF}}_{\text{it}}\right){+\beta}_5\left({\text{EGB}}_{\text{it}}\right)+{\beta}_6\left({\text{ERDE}}_{\text{it}}\right)+{{\beta}_7\left({\text{ETAX}}_{\text{it}}\right)+{\beta}_8\left({\text{GRT}}_{\text{it}}\right)+\varepsilon}_{\text{it}}$$

Equation (1) shows that TCO_2_ is a function of gross domestic product per capita (GDPPC), square of GDPPC, road traffic (RTRF), railway traffic (RLTRF), air traffic (AIRTRF), environmental government budget (EGB), ERDE, environmental taxes (ETAX), and green transport (GRT). Environmental government budget is another important factor in protecting environment quality. TCO_2_ indicates transport-carbon emission ton million, and quadratic term of GDP is used to investigate the transport-carbon emissions (TCO_2_) once economic development achieves a threshold level as EKC hypothesis postulates. It is anticipated to affect TCO_2_ $\left({\beta }_{1}=\frac{\partial {TCO}_{2}}{\partial GDPPC}<or >0\right)$. Road traffic (RTRF) is a crucial and main driving factor in the transport sector, measured by passengers and freights transported via road with the average distance travelled by each passenger and freight, and expected to exert a positive effect on TCO_2_ $\left({\beta }_{2}=\frac{\partial {TCO}_{2}}{\partial RTRF}>0\right)$. Subsequently, railway traffic (RLTRF) is also an important factor in releasing transport-carbon emissions, measured by passengers and freights transported via railway with the average distance travelled by each passenger and freight, and anticipated to exert a positive effect on TCO_2_ $\left({\beta }_{3}=\frac{\partial {TCO}_{2}}{\partial RLTRF}>0\right)$. Likewise, air traffic (ATRF) is a crucial factor in the transport sector, a significant source of TCO_2_ emissions, and expected to exert a positive effect on TCO_2_ $\left({\beta }_{4}=\frac{\partial {TCO}_{2}}{\partial ATRF}>0\right)$.

Hussain et al. (2020a, b) argued that governments allocating national funds for different environmental projects enable government bodies to control the release of greenhouse emissions from several sectors, particularly the transport sector and related industries. Thus, environmental government budget is anticipated to exert a negative effect on transport energy consumption $\left({\beta }_{5}=\frac{\partial {TCO}_{2}}{\partial EGB}<0\right)$. Likewise, ERDE is an important factor for controlling TCO_2_. Wang et al. (2021a, b) and Chishti et al. (2021) proposed that ERDE can reduce TCO_2_ through technological advancement. ERDE is expected to exert a negative effect on TCO_2_ $\left({\beta }_{6}=\frac{\partial {TCO}_{2}}{\partial ERDE}<0\right)$. ETAX is also a signific

ant factor. Li et al. (2021) demonstrated that environmental taxes discourage production entities or companies in releasing a certain amount of CO_2_ emissions. Thus, ETAX is predicted to exert a negative effect on TCO_2_ $\left({\beta }_{7}=\frac{\partial {TCO}_{2}}{\partial ETAX}<0\right)$. In addition, road electric vehicles present a significant effect on TCO_2_. Hu et al. (2021) showed that green transport plays a crucial role in reducing TCO_2_. Green transport is considered a green transport strategy for protecting the environment and anticipated to exert a negative effect on TCO_2_ $\left({\beta }_{8}=\frac{\partial {TCO}_{2}}{\partial GRT}<0\right)$.

2 $${\text{TCO}2}_{\text{it}}={\beta}_0+{{\beta}_1\left({\text{GDPPC}}_{\text{it}}\right)+{\beta}_2\left(\text{GDPPC}_{\text{it}}^2\right)+\beta}_3\left({\text{RTRF}}_{\text{it}}\right)+{\beta}_4\left({\text{RLTRF}}_{\text{it}}\right)+{\beta}_5\left({\text{AIRTRF}}_{\text{it}}\right){+\beta}_6\left({\text{EGB}}_{\text{it}}\right)+{\beta}_7\left({\text{ERDE}}_{\text{it}}\right)+{{\beta}_8\left({\text{ETAX}}_{\text{it}}\right)+{\beta}_9\left({\text{GRT}}_{\text{it}}\right)+\varepsilon}_{\text{it}}$$

We extend the empirical model (Eq. 2) to analyze the nonlinear effect of GDPPC, specifically whether the nexus between TCO_2_ and GDPPC is *U*-shaped or inverted *U*-shaped. The coefficient of squared of GDPPC is predicted to exert a negative effect on TCO_2_ $\left({\beta }_{2}=\frac{\partial {TCO}_{2}}{\partial {GDPPC}^{2}}<0\right)$. We also include the interaction term (GDPPC × ETAX) in the empirical model (Eq. 3) to analyze the joint effect of GDPPC and ETAX on TCO_2_.

3 $${\text{TCO}2}_{\text{it}}={\beta}_0+{{\beta}_1\left({\text{GDPPC}}_{\text{it}}\right)+\beta}_2\left({\text{RTRF}}_{\text{it}}\right)+{\beta}_3\left({\text{RLTRF}}_{\text{it}}\right)+{\beta}_4\left({\text{AIRTRF}}_{\text{it}}\right){+\beta}_5\left({\text{EGB}}_{\text{it}}\right)+{\beta}_6\left({\text{ERDE}}_{\text{it}}\right)+{{\beta}_7\left({\text{ETAX}}_{\text{it}}\right)+{\beta}_8\left({\text{GRT}}_{\text{it}}\right)+{\beta}_9\left({\text{GDPPC}\ast\text{ETAX}}_{\text{it}}\right)+\varepsilon}_{\text{it}}$$

We predict that the coefficient of the interaction term exerts a negative effect on TCO_2_ $\left({\beta }_{9}=\frac{\partial {TCO}_{2}}{\partial GDPPC*ETAX}<0\right)$ in the analysis of the joint effect of GDPPC and ETAX.

4 $${\text{TCO}2}_{\text{it}}={\beta}_0+{\beta}_1\left({\text{INE}}_{\text{it}}\right)+{{\beta}_2\left({\text{GDPPC}}_{\text{it}}\right)+\beta}_3\left({\text{RTRF}}_{\text{it}}\right)+{\beta}_4\left({\text{RLTRF}}_{\text{it}}\right)+{\beta}_5\left({\text{AIRTRF}}_{\text{it}}\right){+\beta}_6\left({\text{EGB}}_{\text{it}}\right)+{\beta}_7\left({\text{ERDE}}_{\text{it}}\right)+{{\beta}_8\left({\text{ETAX}}_{\text{it}}\right)+{\beta}_9\left({\text{GRT}}_{\text{it}}\right)+{\beta}_{10}\left({\text{GDPPC}\ast\text{INE}}_{\text{it}}\right)+\varepsilon}_{\text{it}}$$

In order to estimate the effect of income inequality on transport-carbon emissions, we estimate an empirical model in Eq. (4), which indicates that income equality is investigated along with economic development, traffic, green transport, and environmental expenditures. Income inequality is a crucial factor in environmental domain. It drastically affects the environment quality through multiple economic activities across the countries over the time period (Yan and Yang 2021). Hence, it is anticipated to have negative sign $\left({\beta }_{1}=\frac{\partial {TCO}_{2}}{\partial INE}<0\right)$. In addition, the joint effect of income equality and level of economic development is also examined to analyze the simultaneous impact on transport-carbon emissions. We thus expect to have negative effect on transport-carbon emissions $\left({\beta }_{10}=\frac{\partial {TCO}_{2}}{\partial GDPPC*INE}<0\right)$, which indicates that economic development may reduce transport-carbon emissions through income inequality.

### Data and source

We collect data from different international organizations, such as the Organization for Economic and Development Cooperation (OECD), World Development Indicator (WDI), and Standardized World Income Inequality Database (SWIID). Therefore, data on variables, such as transport-carbon dioxide emissions, road traffic, rail traffic, air traffic, and green transport, are collected from the OECD website, while data on gross domestic product, environmental government budget, research and development, and taxes are collected from WDI, and income inequality data are collected from SWIDD from 2000 to 2020 for 35 OECD countries, namely, the UK, the USA, Portugal, Austria, Poland, Turkey, Latvia, Japan, Denmark, Finland, Czech Republic, Sweden, Switzerland, Chile, Norway, Spain, Korea, Italy, Slovak Republic, New Zealand, Lithuania, Belgium, Slovenia, Mexico, Estonia, Australia, Luxembourg, Hungary, Canada, Netherlands, France, Germany, Greece, Iceland, and Ireland. Furthermore, 11 variables are used in the empirical models. Operational variables and data sources are listed in Table 1.

**Table 1:** Variables and data sources

Variable	Code	Measurement	Source
Transport-carbon emission	TCO_2_	Million ton	OECD
Gross domestic product	GDPPC	GDP per capita ($US million)	WDI
Road traffic	RTRF	Passengers and freights transported via road with average distance travelled by each passenger and freight	OECD
Rail traffic	RLTRF	Passengers and freights transported via railway with average distance travelled by each passenger and freight	OECD
Air traffic	AIRTRF	Passengers and freights transported via air with average distance travelled by each passenger and freight	OECD
Green transport	GRT	Total number of electric vehicles (million)	OECD
Environmental government budget	EGB	Share of percentage of total EGB	WDI
Environmental R&D expenditures	ERDE	Percentage of GDP	WDI
Environmental Tax	ETAX	Percentage of GDP	WDI
Income inequality	INE	Gini coefficient	SWIID

### Estimation methods

EKC theory is discussed in this study to provide an in-depth analysis. The CS-ARDL is a robust method in the presence of cross-dependency, heterogeneity, endogeneity problem, nonstationarity, and misspecification bias (Zeqiraj et al. 2020). We use a common correlated effect mean group (CCEMG) approach for robustness verification.

Countries are interconnected via multiple networks, such as globalization, industrialization, economic integration, and geoeconomics. Hence, the dependency issue will likely occur among the observed variables of sample countries. We use the cross-sectional dependence (CD) method of Pesaran (2004) and scaled LM test. The CD test is expressed as follows:

5 $$\text{CD}=\sqrt{\frac{2\mathrm T}{\mathrm N\left(\mathrm N-1\right)}}\left(\sum\nolimits_{\mathrm i=1}^{\mathrm N-1}\sum\nolimits_{\mathrm j=\mathrm i+1}^{\mathrm N}\tilde{P_{ij}}\right)$$

where ${\stackrel{\sim }{\uprho }}_{\text{ij}}$ is the pairwise correlation of cross-sectional residuals and obtained from augmented Dickey-Fuller (ADF) test. Therefore, time and cross-section dimensions are denoted as “*T*” and “*N*,” respectively. Stationarity among variables is subsequently tested in this study using cross-sectionally augmented CADF and CIPS tests (Pesaran 2007). Thus, the CIPS method is an efficient second-generation panel unit root test for heterogeneity and CD (Pesaran 2007; Moon and Perron 2004; Bai and Ng 2004).

The following equations can be estimated:

6 $$\Delta {\text{CA}}_{\text{i, t}}=\oslash_{\mathrm i}+\oslash_{\mathrm i} {\mathrm Z}_{\mathrm {l, t-1}}+\oslash_{\mathrm i}\overline{\text{CSA}}_{\mathrm {t-1}}+{\Sigma}_{i=0}^\rho \oslash_{\mathrm i\iota}\Delta\overline{\mathrm {CSA}}_{\mathrm {t-1}}+{\Sigma}_{i=0}^\rho\oslash_{\mathrm i\iota}\Delta {\mathrm {CA}}_{\mathrm {i, t-1}}+\mu_{\mathrm {it}},$$

where cross-section averages are denoted as ${\overline{CSA} }_{t-1}$ and $\Delta {\overline{\text{CSA}} }_{\mathrm{t}-1}$. Therefore, the test statistics of CIPS is expressed as

7 $$\tilde{CIPS }= 1/N\sum_{i=1}^{n}{CDF}_{i} .$$

Equation 7 shows that CDF is an indicator of cross-sectional ADF. Error correction-based cointegration method of Westerlund (2007) is also applied to estimate the long-run relationship between observed variables due to its higher suitability than standard methods, such as Pedroni and Kao, and unbiased results over cross-dependency and heterogeneity issues in the estimated model.

The equation can be tested as follows:

8 $${a}_{i}\left(L\right){\Delta \gamma }_{it}={\delta }_{1i}+{\delta }_{2it}+{a}_{i}\left({\gamma }_{it-1}-{\beta }_{i}^{\prime}{x}_{it-1}+{\lambda }_{i}{\left(L\right)}^{\prime}{v}_{it}+{\varepsilon }_{it}\right),$$

where ${\delta }_{1i}={\alpha }_{i}\left(1\right){\phi }_{2i}-{\alpha }_{i}{\phi }_{1i}+{\alpha }_{i}{\phi }_{2i}$, ${\delta }_{2i}=-{\alpha }_{i }{\phi }_{2i}$, and ${\alpha }_{i}$ represent the error correction term. The test statistics is described as

8.1 $${G}_{t}=1/N{\sum }_{i=1}^{N}{~}^{{a}_{i}^{\prime}}\!\left/ \!\!{~}_{SE\left({a}_{i}^{\prime}\right)}\right.$$

8.2 $${G}_{a}=1/N{\sum }_{i=1}^{N}{~}^{{T}_{i}^{\prime}}\!\left/ \!\!{~}_{\left({a}_{i}^{\prime}\right)1}\right.$$

8.3 $${P}_{t}={~}^{{a}^{\prime}}\!\left/ \!\!{~}_{SE\left({a}^{\prime}\right)}\right.$$

8.4 $${a}^{\prime}={P}_{a}/T$$

The error correction parameter $\left({a}^{\prime}\right)$ in Eq. (7) is computed by replacing the value of ${P}_{a}={T}_{{a}^{\prime}}$ (7.4). Hence, the error correction parameter is specified as $\left({a}^{\prime}\right)={P}_{a}/T$ to indicate the ratio of error to be corrected each year in the short run for the disequilibrium case.

The CS-ARDL model of Chudik and Pesaran (2015) is used after detection of cross-dependence and stationarity between parameters to analyze the relationship between observed variables for long and short runs. Therefore, CS-ARDL contains short-run, error correction, cross-sectional mean, and long-run parameters in its framework. This method addresses issues, such as heterogeneity, nonstationarity, and cross-sectional dependence (Zeqiraj et al. 2020; Ahmad et al. 2021). The CS-ARDL model is expressed as follows:

9 $$\Delta {\text{TCO}2}_{\mathrm{i},\mathrm{t}}={{\vartheta }}_{\mathrm{i}}+\sum \nolimits_{\mathrm{j}=1}^{\uprho }{{\vartheta }}_{\text{it}}\Delta {\text{TCO}2}_{\mathrm{i},\mathrm{t}-1}+\sum \nolimits_{\mathrm{j}=0}^{\uprho }{\vartheta }_{ij}^{\prime}{\text{AV}}_{\mathrm{i},\mathrm{t}-1}+\sum \nolimits_{\mathrm{j}=0}^{\uprho }{{\vartheta }}_{\text{it}}\overline{{\mathrm{ Z} }_{\mathrm{t}-\mathrm{j}}}+{\upvarepsilon }_{\text{it}}$$

where $\Delta {\text{TCO}2}_{\mathrm{i},\mathrm{t}}$ is the dependent variable and ${AV}_{i,t-1}$ and ${\overline{Z} }_{t}$ are the independent variable and the cross-section average, respectively.

CCEMG of Pesaran (2006) is applied in this study after CS-ARDL estimation to estimate the robust effect because it allows parameters to be heterogeneous in the long run. Thus, although CS-ARDL imposes a homogeneity restriction, countries are diverse due to the economic system. Analysis of the direction of association between observed variables is lacking despite reliable outcomes obtained by CS-ARDL and CCEMG. Therefore, we apply the causality method to investigate the casual relationship among variables (Dumitrescu & Hurlin, 2012) because it provides the two statistics of test average ($\overline{W }$) and standard normal distribution ($\overline{Z }$). The model can be described as

10 $${Z}_{i,t}= {\alpha }_{i}+\sum_{j=1}^{\rho }{{\beta }^{j}}_{i}{Z}_{i,t-1}+\sum_{j=1}^{\rho }{{\gamma }^{j}}_{i}{T}_{i,t-j} ,$$

where ${\beta }^{j}$ (*j*) and *j* indicate the autoregressive parameter and lag length, respectively.

## Empirical results and discussions

### Descriptive analysis

We document the descriptive statistics in Table 2. The outcomes unveil that mean values (logarithms) and standard deviations of road traffic, rail traffic, air traffic, and GDP are all extremely high, thereby indicating that these variables are highly heterogeneous across OECD countries. Furthermore, EGB and TCO_2_ present high standard deviations of 11.39 and 72.99, respectively. This finding demonstrates that observations are skewed across the sample countries. The small mean value and standard deviation of environmental tax indicated that observations vary within a narrow range over time.

**Table2:** Descriptive statistics

Variable	Obs	Mean	S.D	Min	Max
TCO_2_	735	1.9668	1.8633	1.0492	2.9350
RTRF	735	4.9272	5.7387	0.0312	6.7168
RLTRF	735	4.7740	5.2600	0.2651	6.0569
AIRTRF	735	4.4710	5.2324	0.4422	6.3269
GDP	735	5.8234	6.4698	0.2043	7.3541
EGB	735	1.4098	1.0568	0.6213	1.7704
ERDE	735	0.3961	0.3209	1.3010	1.2469
ETAX	735	0.9829	0.9244	0.3213	0.2345
ROAD-ELECT	735	1.3079	0.9100	0.2218	5.8143
RAIL-ELECT	735	1.0098	0.3645	0.5228	4.5072
AIR-ELECT	735	1.0029	0.1234	0.4322	0.2343
INE	735	3.445	0.171	3.118	3.968

All values are logarithms

Source: authors’ calculations

Additionally, the cross-dependency of the observed variables is investigated. Thus, the presence of CD is essential to be detected for analyzing the empirical models. According to Pesaran (2004), ordinary econometric methods characteristically failed to overcome the bias in the panels due to the presence of CD. Pesaran’s CD and scaled LM test estimate are presented in Table 3. The upshots indicate that CD is supported by the absolute mean value (0.696–107.619). Hence, the highly significant outcomes of the Pesaran’s CD and scaled LM tests for all observed variables, which signals those variables, contain CD. Notably, countries are becoming increasingly entangled due to globalization. The CD test results are significantly projected in the model. Some possible changes in the observed variables may affect on other countries. For instance, OECD countries show high dependence and statistically significant variables, such as economic development, green transport, and transport emissions, along with higher magnitudes compared with others because international borders are nonexistent with carbon emissions, which travel freely and affect the other countries. Likewise, road, railway, and air traffic as well as government budget have shown high dependence on economies.

**Table 3:** Cross-sectional dependence

Variable	Pesaran CD		Pesaran-scaled LM
	CD test	abs(corr)	CD test
TCO_2_	112.153^a^	0.90	131.633^a^
RTRF	25.119^a^	0.20	221.433^a^
RLTRF	46.43^a^	0.40	144.228^a^
AIRTRF	29.434^a^	0.26	120.938^a^
GDP	56.477^a^	0.16	111.433^a^
EGB	8.149^a^	0.30	105.122^a^
ERDE	3.186^a^	0.08	97.722^a^
ETAX	34.142^a^	0.55	133.541^a^
GDP2	100.157^a^	0.90	180.232^a^
GRT	124.211^a^	0.78	190.302^a^
INE	10.231^a^	0.56	90.233^a^

Author’s calculations

Note: Table 2 reveals the estimate of cross-dependency (CD) test of Pesaran CD and Pesaran-scaled LM of observed variables of 35 OECD countries

^a^, ^b^, and ^c^ show statistical significance at 1%, 5%, and 10%, respectively.

Values in parenthesis are standard errors.

We use the second-generation panel unit root test (CIPS and CADF) of Pesaran (2007) to investigate the order of integration and demonstrate that CIPS is cross-sectionally unbiased. The order of integration is a unique factor to consider in estimation techniques. The CIPS and CADF results are summarized in Table 4. Findings suggest that entire variables are stationary at 1(1) and follow a mixed order of integration. The use of the CS-ARDL framework is required when CD and a mixed order of integration exist. The long-run relationship in the models has been investigated by using the Westerlund cointegration approach. The upshots in Table 5 demonstrate the presence of a long-run relationship in the models. In addition, the *P* value in the models can be used to calculate the error correction (EC). Hence, the EC parameter is $\left({\alpha }^{\prime}=\frac{{P}_{\alpha }}{T}\right)$ =$\frac{-2.76}{18}=-0.153$ for model 1, $\frac{-4.893}{18}=-0.272$ for model 2, and $\frac{-2.854}{18}=-0.158$ for model 3. Disequilibrium in the short run becomes stable in the long run because approximate errors of 19.43% between TCO_2_ and its determinants are corrected each year.

**Table 4:** Panel unit root

Variable	Cross-sectionally augmented IPS (CIPS)		Cross-sectionally augmented Dickey-Fuller (CADF)	
	Level	First-difference	Level	First-difference
TCO_2_	− 2.198	− 1.132^a^	− 4.279^a^	− 10.081^a^
RTRF	− 0.771	− 0.232^b^	9.961^c^	4.678^a^
RLTRF	− 1.198	− 1.327^a^	1.823	− 0.599^a^
AIRTRF	− 0.090 ^a^	-	10.219	6.466^a^
GDP	− 0.152^a^	-	11.296	10.567^a^
EGB	− 2.314^b^	− 1.132^a^	− 1.376^c^	− 7.157^a^
ERDE	− 0.230 ^b^	− 0.232^c^	12.114^c^	3.925^b^
ETAX	− 1.554	− 1.322c	2.258	− 2.340^a^
GDP2	− 2.310	0.123^c^	− 1.309^c^	− 8.049^a^
GRT	− 0.516^b^	− 0.231^c^	12.714	3.921^b^
INE	− 0.102^a^	− 0.231^b^	− 0.154^a^	− 0.112^a^

Author’s calculations

CIPS, CADF

^a^, ^b^, and ^c^ show statistical significance at 1%, 5%, and 10%, respectively.

Values in parenthesis are standard errors.

**Table 5:** Cointegration test

Variable	Model 1	Model 2	Model 3	Model 4
Gt	− 2.557^a^ (− 4.123)	− 1.440 (0.323)	− 1.635^a^ (0.083)	− 1.215^a^ (0.033)
Ga	− 2.636^a^ (0.032)	− 3.354 (4.322)	− 3.983^b^ (0.321)	− 2.183^b^ (0.121)
Pt	− 1.093^a^ (− 0.054)	− 6.091^a^ (− 0.482)	− − 4.259^a^ (− 0.043)	− 3.159^a^ (− 0.043)
Pa	− 2.761^a^ (0.483)	− 4.893^a^ (− 1.212)	− 2.854^a^ (0.321)	− 1.054^a^ (0.011)

Author’s calculations.

Westerlund cointegration test.

^a^, ^b^ and ^c^ show statistical significance at 1%, 5% and 10%, respectively.

Values in parenthesis are standard errors.

### Empirical results

#### 4.2.1 Effect of economic development, traffic, and environmental expenditures

Considering the impact of economic development and traffic on transport-carbon dioxide emissions, the coefficient of GDPPC is a positive and statistically significant for TCO_2_ emissions. This implies that a 1% increase in economic development (GDPPC) upsurges 26.4% and 38.8% of transport-carbon dioxide emissions (TCO_2_) on average in the short and long runs, respectively. Notably, the occurrence of economic activities is relatively less in the short run than that in the long term and outputs of inputs are difficult to quantify, particularly carbon emissions released from the transport sector. However, large economic activities in the long run are accounted in terms of inputs and outputs over a time period with available resources. Economic development and affecting factors require a specific time period for quantification. Consequently, long-run emissions are considered more than short-run ones. The negative impact of the coefficient of GDPPC^2^ on TCO_2_ emissions implied that TCO_2_ emission reduction of 12.1% is due to the 1% increase in GDPPC^2^ in the long run. Short-run outcomes showed that an increase in economic development significantly reduces transport-carbon dioxide emissions and its likelihood to occur is analytically tested through the EKC hypothesis. This finding implied that a 1% increase in GDPPC^2^ diminishes 33.3% of TCO_2_ emissions in the short run. Similar to other studies, the issue of an inverted *U*-shaped curve representing the relationship between carbon emissions and economic development indicated that economic development increases carbon dioxide emissions in the short and long runs through several economic activities at the initial stage (Ahmad et al. 2020; Zeqiraj et al. 2020).

In addition, the traffic (road, railway, and air) is a major source of carbon dioxide emissions that pollute the environment. Therefore, the coefficients of traffic show the significant effect on TCO_2_ emissions. Specifically, the coefficient of road traffic (RTRF) causes the carbon dioxide emissions, which indicates that a 1% increase in RTRF upsurges the TCO_2_ emissions by 47.17% in the long run. On the contrary, in the short run, TCO_2_ emissions increased by 64.7% due to a 1% change in road traffic. Similarly, the coefficient of railway (RLTRF) shows that TCO_2_ emissions increase by 33.68% and 30.5% due to the 1% change in RLTRF on average in long and short runs, respectively. In the contrast, air traffic (ATRF) has larger impact on TCO_2_ emissions at 70.13% than RTRF and RLTRF in the short run in the OECD countries, while TCO_2_ emissions increase by 32.46% due to a 1% change in ATRF. However, our results are drastically different from other findings, particularly in the short run, because the OECD countries are the attractive places for trade and tourism activities. Thus, the large traffic volume induces to release the carbon dioxide emissions under such circumstances.

Additionally, the negative and significant coefficients of environmental government budget (EGB) in Table 6 indicate that a 1% increase in EGB reduces TCO_2_ around 146% (linear), 13.2% (nonlinear), and 2.1% (interaction) in the short run. On average, TCO_2_ decrease by 28.6%, 1.7%, and 22.1% in all cases due to EGB in the long run. These findings unveil those existing environmental regulations of the OECD countries are effective for environmental protection, especially in terms of TCO_2_ emissions. Yang et al. (2020) and Fan et al. (2020) argue government budgets allocated to the environmental protection and quality have remarkable impact on the CO_2_ emissions and are driving factors against environmental challenges. However, lack of fund may increase the difficulties in identifying environmental vulnerabilities. Consequently, the government budget assists relevant agencies or entities in addressing the issues. In this situation, the OECD countries should allocate the government budgets related to environment for enhancing the environment quality.

**Table 6:** CS-ARDL estimations

Variable	Dependent variable:${TCO2}_{i,t}$								
	Long run					Short run			
	Linear	Nonlinear	EDV interaction	INE interaction		Linear	Nonlinear	EDV interaction	INE interaction
${GDPPC}_{i,t}$	0.421^a^ (0.115)	0.411^a^ (0.111)	0.327^b^ (0.160)	0.345^a^ (0.030)	${\Delta GDPPC}_{i,t}$	0.338^a^ (0.152)	0.347^b^ (0.134)	0.107^a^ (0.035)	0.222^a^ (0.002)
${GDPPC}_{i,t}^{2}$	-	− 0.121^c^ (0.120)	-	-	$\Delta {GDPPC}_{i,t}^{2}$	-	− 0.330^c^ (0.192)	-	-
${RTRF}_{i,t}$	0.542^c^ (0.344)	0.431^b^ (0.231)	0.442^c^ (0.252)	0.432^b^ (0.102)	${\Delta \text{RTRF}}_{i,t}$	1.571^c^ (0.877)	0.104^a^ (0.032)	0.267^c^ (0.140)	0.132^a^ (0.032)
${RLTRF}_{i,t}$	0.311^b^ (0.225)	0.369^b^ (0.067)	0.329^a^ (0.128)	0.201^c^ (0.061)	${\Delta \text{RLTRF}}_{i,t}$	0.242^b^ (0.186)	0.348^b^ (0.235)	0.325^b^ (0.124)	0.102^c^ (0.120)
${AIRTRF}_{i,t}$	0.138^b^ (0.044)	0.278^a^ (0.144)	0.558^b^ (0.310)	0.101^c^ (0.162)	${\Delta \text{AIRTRF}}_{i,t}$	1.216^b^ (0.202)	0.355^c^ (0.312)	0.553^b^ (0.181)	0.432^c^ (0.182)
${EGB}_{i,t}$	− 0.221 (0.018)	− 0.017^b^ (0.089)	− 0.286^c^ (0.499)	0.021 (1.023)	${\Delta \text{EGB}}_{i,t}$	− 0.02^b^ (0.565)	− 0.131^c^ (0.606)	− 1.461^a^ (0.558)	− 0.032^c^ (0.032)
${ERDE}_{i,t}$	− 0.028^a^ (0.010)	− 0.018^a^ (0.012)	− 0.581^b^ (0.243)	− 0.031^b^ (0.032)	${\Delta \text{ERDE}}_{i,t}$	− 0.217^a^ (0.012)	− 0.321^a^ (0.032)	− 0.031^a^ (0.02)	− 0.021^b^ (0.032)
${ETAX}_{i,t}$	− 0.021^a^ (0.012)	− 0.038^b^ (0.432)	− 0.468^a^ (0.010)	− 0.014^b^ (0.021)	${\Delta \text{ETAX}}_{i,t}$	− 0.021^b^ (0.023)	− 0.028^b^ (0.024)	− 0.55^b^ (0.032)	− 0.010^a^ (0.012)
${GRT}_{i,t}$	− 0.432^a^ (0.021)	− 0.412^a^ (0.032)	− 0.359^a^ (0.012)	− 0.321^a^ (0.120)	${\Delta \text{GRT}}_{i,t}$	− 0.281^b^ (0.042)	− 0.351^a^ (0.024)	− 0.234^a^ (0.012)	0.102^a^ (0.130)
${INE}_{i,t}$	-	-	-	− 0.321^a^ (0.041)	${\Delta \text{INE}}_{i,t}$	-	-	-	− 0.210^a^(0.021)
$$\left(\begin{array}{c}{GDPPC}_{i,t}\\ \times {ETAX}_{i,t}\end{array}\right)$$	-	-	− 0.161^c^ (0.134)	-	$$\Delta \left(\begin{array}{c}{GDPPC}_{i,t}\\ \times {ETAX}_{i,t}\end{array}\right)$$	-	-	− 0.133^b^ (0.216)	-
$$\left(\begin{array}{c}{GDPPC}_{i,t}\\ \times {INE}_{i,t}\end{array}\right)$$	-	-	-	0.021^a^ (0.023)	$$\Delta \left(\begin{array}{c}{GDPPC}_{i,t}\\ \times {INE}_{i,t}\end{array}\right)$$	-	-	-	0.013^b^ (0.062)
-	-	-	-	-	$ECT\left(-1\right)$	− 0.059^a^ (0.029)	− 0.180^b^ (0.079)	− 0.243^a^ (0.051)	− 0.129^a^ (0.040)

^a^, ^b^, and ^c^ show statistical significance at 1%, 5%, and 10%, respectively.

Values in parenthesis are standard errors.

Authors’ estimations.

Considering the effect of environmental research and development expenditures (ERDE), the coefficient indicates a substantial negative effect on TCO_2_ emissions over the time period. Therefore, a 1% increase of ERDE in the short run lowers the TCO2 by 3.1% (linear), 32.1% (nonlinear), and 21.7% (interaction). On the contrary, the TCO_2_ is decreased by 58.1% in the long run as a result of 1% change in the ERDE. These findings are consistent with those of Wang et al. (2021a, b) and Chishti et al. (2021), wherein ERDE exerts a significant effect on TCO_2_ because the former findings stimulate the development of new technologies for addressing the environmental problems.

Furthermore, the ERDE square coefficient illustrates that a 1% increase in ERDE square reduces the TCO_2_ approximately 32.3% (linear), 12.8% (nonlinear), and 43.2% (interaction) in the short term. The negative association between ERDE square and TCO_2_ can also be observed in the long run for sample countries. Findings suggest that TCO_2_ may be lowered by technical innovation and environmental economic strategies after achieving a certain level of R&D, and the countries are taking serious consideration regarding the environment to adopt the alternative strategies, such as green transportation financed with green money (Kong et al. 2021; Song et al. 2021).

Thenceforward, we investigate the impact of environmental taxes on TCO_2_ emaciations; the coefficients indicate that there are negative and significant correlation with environmental taxes and carbon emission released by the transport sector in the sample countries. Thus, empirical evidence endorses those environmental taxes cause a 55% (linear), 2.8% (nonlinear), and 2.1% (interaction) reduction in the TCO_2_. Other studies, e.g., Hao et al. (2021), Ma et al. (2021), and Zhai et al. (2021), confirm our findings, and suggest that environmental taxes have significant impact on CO_2_ emissions in declination. The taxes are sources of revenue for government organizations, and they use this fund at large-scale programs, such as sustainable development, agriculture, industrial expansion, and infrastructure (Arvin et al. 2021). Subsequently, effect of green transportation is also investigated in the current analysis; thus, the coefficient of green transport is negatively correlated with TCO_2_. Empirical evidence shows that a 43.2% decrease in TCO_2_ is due to a 1% increase green transport in the short run. On the contrary, long-run outcomes unveil that a 1% change in green transport decreases the transport-carbon emission by 76.5% over the time period in sample countries. Moreover, the inverted *U*-shaped relationship between TCO_2_ and GDP indicates that TCO_2_ may be lowered using enhanced transportation and environmental taxes when a certain level of GDP is achieved. Thus, the OECD countries gradually transfer their resources away from diesel automobiles and toward electric vehicles. Our findings are consistent with those of Xu et al. (2021) and Zhang and Hanaoka (2021), both of which suggest that electric cars have significant impact on TCO_2_ emissions as an alternative mode of transportation.

Interestingly, the interaction term between GDPPC and ETAX is negative and statistically significant in the long run. Therefore, the mutual effect of GDPPC and ETAX reduces TCO_2_, indicating that governments allocate their budgets during implementing the environmental levies to reduce CO_2_ emissions. More analytically, a 1% increase in interaction term (GDPPC*ETAX) reduces transport-carbon emissions by 16.1% and 13.3% in the long run and short run, respectively. This suggests that countries can re-allocate the resources in long term rather than short run. Because, in the short-run period, countries cannot vary the resources or re-allocate the resources concern over environmental quality. In addition, upshots reveal that marginal transport-carbon emissions of environmental tax $\left(\partial TCO2/\partial ETAX\right)$, (while keeping constant other explanatory variables in the current estimated model) is influenced by economic development. On the contrary, economic development also affects the marginal TCO_2_ through environmental taxes. However, this influence is diverse in relationship direction. Hence, GDPPC and ETAX affect the transport-carbon emissions simultaneously in the OECD countries, because of the better economic system that indicates well-documented economies. In contrast, some other scholars (e.g., Wang et al. 2020a, b, c; Wang et al. 2020a, b, c) analyzed the relationship between transport-carbon emissions and economic decoupling. Their argument suggests that transport-carbon intensity overall declined due to polarization trend across the panel. Furthermore, weak, recessive, and expansive negative decoupling substantially affects the transport-carbon emissions. Subsequently, transport-carbon emissions substantially reduced due to economic development in the Belt and Road countries. Besides, Wang et al. (2021a, b) and Wang et al. (2020a, b, c) argue that logistic infrastructure (transport) has long-run relationship with economic development, indicating that transport infrastructure increases the economic development and vice versa. Moreover, carbon emissions released by transport sector are stable in the developed countries; however, developing countries increased the level of transport-carbon emissions within specific time period.

#### Effect of income inequality

After analyzing the impact of level of economic development, traffic, and environmental expenditures on transport emissions, now, we estimate the impact of income inequality along with variables which are observed in the previous model. Thus, the outcomes are provided in Table 6, column 5 and column 10. The results unveil that the coefficient of income inequality is positive and statistically significant in the both long run and short run. Empirical evidence shows that 1% increase in income inequality decreases transport-carbon emissions by 32.1% in the long run. This suggests that income inequality stimulates the economic activities, because, small portion of income is possessed by a few people or entities, which can be invested in productive projects within or across the countries. Moreover, higher income class has the potential to innovate new technology related to transport by spending on research and development concern over environmental quality (Hussain et al. 2021; Wang et al. 2020a, b, c; Zhang and Zhang 2021; Yan and Yang 2021). The short-run outcomes also confirm income inequality is negatively associated with transport-carbon emissions across the OECD countries. This implies that a 21% decrease in transport-carbon emissions is due to a 1% change in income inequality, suggesting that resources are slightly shifted to innovate a new technology related to transport concern over emissions with short period of time. Consequently, long run has larger impact transport-carbon emission rather than short run. Former studies (e.g., Song 2021; Poi et al. 2021; Feng et al. 2021; Zhang and Zhang 2021) also confirm our findings that income inequality negatively associated with carbon emissions. However, their research concentrates merely on total carbon dioxide emissions rather than specific sector.

More broadly, considering the joint effect of income inequality and economic development on transport-carbon emissions, the coefficient of interaction term (GDPPC*INE) is positive and statistically significant for the TCO_2_ in the time of period. The outcomes unveil that a 1% increase in interaction of economic development and income inequality upsurges transport-carbon emission by 2.1% and 1.3% in the long run and short run, respectively. This means that level of economic development increases the transport-carbon emissions through income inequality. More analytically, the marginal impact of economic development $\left(\frac{\partial TCO2}{\partial GDPPC}>0\right)$, while keeping constant other explanatory variables, is positive that increases in total carbon emission released by transport sector. However, income inequality drastically influences the marginal impact of economic development on transport-carbon emissions. For instance, on the contrary, the marginal impact of income inequality $\left(\frac{\partial TCO2}{\partial INE}>0\right)$ is also found to be positive factor for TCO_2_. This suggests that economic development positively influences the marginal transport emission of income inequality. Specifically, an increase in level of economic development may upsurge income inequality that causes carbon emissions through different channels, particularly transport sector and vice versa.

### Robustness check

This study also assesses the robust effect in the current model to check the consistent outcomes, which are obtained from CS-ARDL method. To do so, we employ the CCMEG technique that estimates correlation with unobserved variables and error term with explanatory variables. Therefore, this model can be used to correlate the unobserved factors, such as economic development, income inequality, transportation investment, energy consumption, freight and passenger volume, and CO_2_ emissions from other sectors. The outcomes are provided in Table 7, which unveil those outcomes obtained from CS-ARDL are consistent and reliable. Besides, we analyze the causal relationship between the transport-carbon emissions and its influencers (which are incorporated in the current models) by using Granger causality test suggested by Dumitrescu and Hurlin (2012). Therefore, the outcomes are summarized in Table 8, and suggest that road traffic and economic development have bidirectional relationship with transport-carbon emissions. This means that any policy shock in transport-carbon emissions may affect the both road traffic and economic development, and vice versa. On the contrary, one-way casual effect of transport-carbon emissions with income inequality, railway and air traffic, environmental expenditure, and green transport suggests that any policy shock may not affect the aforementioned variables across the countries over the time.

**Table 7:** Robustness check

	Model 1	Model 2	Model 3	Model 4
${GDPPC}_{i,t}$	0.0295^a^ (0.021)	0.040^a^ (0.032)	3.245^a^ (0.012)	1.145^a^ (0.02)
${GDPPC}_{i,t}^{2}$	0.0428^b^ (0.121)	0.022^a^ (0.032)	0.019^a^ (0.123)	-
${RTRF}_{i,t}$	0.075^a^ (0.231)	0.096^a^ (0.432)	− 0.112^b^ (0.234)	-0.012^b^ (0.034)
${RLTRF}_{i,t}$	0.089^a^ (0.043)	2.210 (0.843)	3.041^a^ (0.542)	0.041^a^ (0.042)
${AIRTRF}_{i,t}$	0.0382^b^ (0.074)	0.124^a^ (0.065)	0.132^a^ (0.431)	0.032^a^ (0.031)
${EGB}_{i,t}$	-	0.425^a^ (0.043)	-	0.025^a^ (0.013)
${ERDE}_{i,t}$	-	− 2.1705^b^ (0.432)	-	− 0.163^b^ (0.012)
${ETAX}_{i,t}$	-	0.314^a^ (0.102)	-	0.011^a^ (0.001)
${GRT}_{i,t}$	-	-	0.546^a^ (0.103)	0.546^a^ (0.103)
$$\left(\begin{array}{c}{GDPPC}_{i,t}\\ \times {ETAX}_{i,t}\end{array}\right)$$	-	-	− 0.031^a^ (0.112)	-
$$\left(\begin{array}{c}{GDPPC}_{i,t}\\ \times {INE}_{i,t}\end{array}\right)$$	-	-	-	0.006^a^ (0.001)
Observations	630	630	630	630
*R*-squared	0.639	0.316	0.355	0.403
Number of groups	35	35	35	35

^a^, ^b^, and ^c^ show statistical significance at 1%, 5%, and 10%, respectively.

Values in parenthesis are standard errors.

**Table 8:** Panel causality test results

Null hypothesis	*W*-statistics	Zbar-statistics	Prob	Conclusion
TCO_2_ $\nleftrightarrow$ RTRF	1.123^a^	0.243	0.000	
RTRF $\nleftrightarrow$ TCO_2_	1.137^a^	0.232	0.000	RTRF $\leftrightarrow$ TCO_2_
TCO_2_ $\nleftrightarrow$ RLTRF	1.443	0.733	0.234	
RLTRF $\nleftrightarrow$ TCO_2_	1.454^a^	0.673	0.000	RLTRF $\to$ TCO_2_
TCO_2_ $\nleftrightarrow \text{AIRTRF}$	2.1230	0.3638	0.716	
$\text{AIRTRF}\nleftrightarrow$ TCO_2_	1.4327^a^	− 1.6781	0.003	AIRTRF $\to$ TCO_2_
TCO_2_ $\nleftrightarrow$ EGB	2.006	0.0177	0.985	
EGB $\nleftrightarrow$ TCO_2_	3.3591^a^	4.0204	0.001	EGB $\to$ TCO_2_
TCO_2_ $\nleftrightarrow$ ERDE	1.343	0.393	0.089	
ERDE $\nleftrightarrow$ TCO_2_	1.543^a^	0.493	0.003	$ERDE\to$ TCO_2_
TCO_2_ $\nleftrightarrow \text{GDPPC}$	3.4939^a^	4.4189	0.000	
$\text{GDP}\nleftrightarrow$ TCO_2_	6.9714^a^	14.7057	0.000	$\text{GDPPC}\leftrightarrow$ TCO_2_
TCO_2_ $\nleftrightarrow \text{GRT}$	2.1929^a^	14.189	0.230	
$\text{GRT}\nleftrightarrow$ TCO_2_	3.2714^a^	11.7057	0.000	$\text{GRT}\to$ TCO_2_
TCO_2_ $\nleftrightarrow INE$	1.1291^a^	11.119	0.130	
$INE\nleftrightarrow$ TCO_2_	0.2114^a^	10.1057	0.000	$INE\to$ TCO_2_

Author’s calculations.

“$\to$” indicates one-way causality, while “$\leftrightarrow$” two-way causality between the variables.

^a^, ^b^, and ^c^ show statistical significance at 1%, 5%, and 10%, respectively.

## Conclusion

This paper concentrates on the environmental issues, especially carbon dioxide emissions released by the transport sector in the OECD countries period from 2000 to 2020. In addition, this study evaluates the role of economic development, income inequality, green transport, and environmental expenditures. To do so, we use an advanced econometric approach, e.g., second-generation cross-sectional autoregressive distributed lag suggested by Chudik and Pesaran (2015), for long-run and short-run relationship between transport-carbon emission and its influencers. Besides, cointegration test suggested by Westerlund (2007) is also employed for long-run estimation.

We document the empirical findings, which suggest that there is a long-run cointegration relationship that exists between the observed variables over the time. The outcomes obtained from CS-ARDL unveil that road, railway, and air traffic significantly upsurge the transport-carbon emissions by 54.2%, 31.1%, and 13.8%, respectively, in the long run. More precisely, a large volume of road traffic drastically grows transport-carbon emissions compared with railway and air, because of the usage of fossil fuel vehicles, such as buses, cars, and motorbikes. Besides, economic development also initially (e.g., positive marginal impact of economic development) pollutes the environment through the contribution of transport-carbon emissions approximately 42% across the countries. After reaching at specific level, diminishing the marginal impact of economic development declines the total transport-carbon emissions by 12.1%, which indicates that there is an inverted *U*-shaped relationship that exists between transport-carbon emissions and economic development. Analytically, this decline in transport-carbon emissions due to economic development also may via environmental expenditures. More interestingly, income inequality has remarkable negative impact on transport-carbon emissions. However, the outcomes further suggest that the joint effect of income inequality and economic development reduces the transport-carbon emissions across the countries.

Additionally, environmental government budget is found to be essential factor that has remarkable negative effect on environment; empirically, it reduces transport-carbon emissions approximately 22.1% by one unit change in terms of million dollars through research and development channel. Subsequently, environmental taxes also drastically impact, and reduce the transport-carbon emission by 2.1% in the long run. Besides, green transport has remarkable impact on transport-carbon emissions, for instance, transport-carbon emissions can be declined around 43.2% by one unit change in green transport across the countries. More precisely, electric vehicles are prime alternative strategies to mitigate the carbon pollute environment, especially polluted by transport sector. Finally, the causality upshots (D&H) demonstrate that any policy that focuses on the economic development, road traffic, or environmental taxes has considerable impact on transport-carbon emissions, and vice versa. Moreover, any direction affecting transportation railway and air traffic, environmental government budgets, environmental taxes, green transport, and income inequality indicates significant impact on transport-carbon emissions. On the contrary, most factors remained unaffected by transport-carbon emissions-related policies.

## Policy implications

This study suggests the following policy recommendations to transportation experts/economists, urban planners, and transport modelers. (1) Environmental degradation can be prevented only, if the policymakers implement the extreme measures to reduce the traffic movement within the countries. Furthermore, transport institutes must regulate the flow of traffic on the roads. Traffic movement plan can potentially cut the CO_2_ emissions from the transportation sector by around 33% on average. (2) Government entities or transportation experts/policymakers can improve the environment by implementing the strategies in order to reduce the widespread automobile use. (3) The countries should enhance their environmental budgets approximately 4% to reduce the CO_2_ emissions. An improvement in environmental budgetary plan may be established to maintain the nonrenewable resources. (4) The countries should produce the additional green transportation, such as electric road and rail vehicles. (5) Policies related to environmental taxation and R&D expenditures must be formulated to achieve best output level in the long run. (6) Urban planners must design transportation infrastructure that allows the traffic to freely move at the city level to prevent the environment from CO_2_ emissions. (7) Transportation modelers can create a policy by considering the demand for and supply of green transportation products, such as electric automobiles. (8) Experts in transportation (producers) should minimize the manufacturing or production costs of railway and road vehicles, which waste a substantial amount of energy during the movements as well as impose restriction the traffic movements inside metropolitan areas to impede the contribution of transportation to carbon emissions.

This study describes the following limitations. The scope of this research is confined to OECD countries. Furthermore, only road, rail, and air traffic; economic development; income inequality; green transportation; and environmental expenditures are considered when analyzing the effect on transport-carbon emissions because travel is a major source of emissions, which exhibits that passengers and freight move over the long distances by the road, rail, and air. Consequently, traffic has received more research attention than other aspects. Furthermore, electric vehicles are used for green transportation to address environmental concerns. This work may be expanded in future investigations by including the environmental technology, population agglomeration, and institutional quality. Investigating implications of green financing, transportation, and technology in emerging economies can provide policymakers and practitioners increasingly precise information.

## Data Availability

The datasets used and/or analyzed during the current study are available from the corresponding author on reasonable request.
